# Prediction of pancreatic fistula after pancreatoduodenectomy by preoperative dynamic CT and fecal elastase-1 levels

**DOI:** 10.1371/journal.pone.0177052

**Published:** 2017-05-11

**Authors:** Jung-Hyun Kang, Joon Seong Park, Jeong-Sik Yu, Jae-Joon Chung, Joo Hee Kim, Eun-Suk Cho, Dong Sup Yoon

**Affiliations:** 1 Department of Radiology, Yonsei University College of Medicine, Gangnam Severance Hospital, Seoul, Korea; 2 Department of Surgery, Yonsei University College of Medicine, Gangnam Severance Hospital, Seoul, Korea; University of Nebraska Medical Center, UNITED STATES

## Abstract

**Objective:**

To validate preoperative dynamic CT and fecal elastase-1 level in predicting the development of pancreatic fistulae after pancreatoduodenectomy.

**Materials and methods:**

For 146 consecutive patients, CT attenuation values of the nontumorous pancreatic parenchyma were retrospectively measured on precontrast, arterial and equilibrium phase images for calculation of enhancement ratios. CT enhancement ratios and preoperative fecal elastase-1 levels were correlated with the development of pancreatic fistulae using independent t-test, logistic regression models, ROC analysis, Youden method and tree analysis.

**Results:**

The mean value of enhancement ratio on equilibrium phase was significantly higher (p = 0.001) in the patients without pancreatic fistula (n = 107; 2.26±3.63) than in the patients with pancreatic fistula (n = 39; 1.04±0.51); in the logistic regression analyses, it was significant predictor for the development of pancreatic fistulae (odds ratio = 0.243, p = 0.002). The mean preoperative fecal elastase-1 levels were higher (odds ratio = 1.003, p = 0.034) in the pancreatic fistula patients than other patients, but there were no significant differences in the areas under the curve between the prediction values of CT enhancement ratios and fecal elastase-1 combined and those of CT enhancement ratios alone (P = 0.897, p = 0.917) on ROC curve analysis. Tree analysis revealed that the CT enhancement ratio was more powerful predictor of pancreatic fistula than fecal elastase-1 levels.

**Conclusion:**

The preoperative CT enhancement ratio of pancreas acquired at equilibrium phase regardless of combination with fecal elastase-1 levels might be a useful predictor of the risk of developing a pancreatic fistula following pancreatoduodenectomy.

## Introduction

The rate of mortality following pancreatoduodenectomy (PD), with or without pylorus preservation, has significantly declined over the last decades [1,2]. However, the post-PD morbidity rate is still high (30–60%) [1–4]. Postoperative pancreatic fistula (POPF) following PD is the main cause for post-PD morbidity, and results in longer hospital stays, increased costs of hospitalization and treatment, or even death [5–8]. Therefore, identifying patients at high risk for POPF is important for decreasing the post-PD morbidity and improving the clinical outcome. In previous studies, several factors including a soft pancreas, pancreatic duct size, fatty pancreas and obesity were recognized as the risk factors for POPF [5–11]. Of these factors, the presence of a ‘soft’ or ‘normal’ pancreatic texture is the most widely accepted risk factor for developing a pancreatic fistula following PD [5,9,12]. On the other hand, pancreatic fibrosis with a ‘firm’ or ‘hard’ pancreatic texture is thought to decrease the risk of POPF. Previous studies have reported that the rate of POPF is low in the fibrotic pancreases with firm parenchyma [9–11,13].

Therefore, the preoperative assessment of pancreatic fibrosis might be helpful not only in predicting the development of pancreatic fistulae after PD, but also in preoperative patient counseling and postoperative management. Thus far, only a few studies have evaluated whether the degree of pancreatic fibrosis can be reliably estimated and quantified radiologically [14–18]. On dynamic computed tomography (CT) or magnetic resonance imaging (MRI), a fibrotic pancreas with autoimmune pancreatitis or chronic pancreatitis shows delayed enhancement with a slow increase followed by a slow decline or a plateau, whereas a normal soft pancreas shows rapid enhancement and a rapid decrease [14–18]. On the other hand, the normal exocrine pancreatic function of the ‘soft’ pancreatic tissue, as compared to that of the ‘hard’ fibrotic pancreatic tissue in patients with chronic pancreatitis, has been reported as one of the risk factors for POPF [19,20]. Fecal elastase-1 is a pancreas-specific enzyme and has been proposed as a suitable marker for pancreatic insufficiency [21–23]. We hypothesized that the delayed phase images of dynamic CT, which can better reveal parenchymal fibrosis than those acquired at the earlier phases, and the combined evaluation of the preoperative CT data and fecal elastase-1 levels could provide more accurate information for predicting the development of POPF. The aim of this study was to evaluate the stand-alone and combined abilities of preoperative dynamic CT and fecal elastase-1 levels to predict the development of pancreatic fistulae after pancreatoduodenectomy.

## Materials and methods

This retrospective study was approved by the Gangnam Severance Hospital institutional review board (IRB) for clinical studies, and the requirement for informed patient consent was waived.

### Patients and clinical data collection

Among the 347 consecutive patients who underwent PD with or without pylorus preserving surgery between January 2006 and March 2015, 148 patients had medical records of POPF and pre-operative dynamic CT data available for retrospective analysis. During the preliminary review of CT, two of the patients were excluded due to the mostly atrophic parenchyma to draw a region of interest (ROI), which was of at least 3-mm diameter on the CT images. Of the remaining 146 patients recruited for the data analysis, 82 (56%) were male and 64 (44%) were female; the mean age of the patient population was 62 years. Fifteen patients had benign diseases (intraductal papillary mucinous neoplasm, 9; solid pseudopapillary tumor, 2; benign neuroendocrine tumor, 1; serous cystadenoma, 1; chronic pancreatitis, 1; and choledochal cyst, 1), and 131 patients had malignant diseases (malignant pancreatic diseases, 45; common bile duct cancer, 45; ampullary cancer, 40; and duodenal cancer, 1). Preoperative dynamic CT was performed 1 to 144 days (median 12 days) before pancreatoduodenectomy; besides the six patients (one benign neuroendocrine tumor, and five intraductal papillary mucinous neoplasms; 61 to 144 days), time intervals between the dynamic CT and the operation were within 35 days for all other patients. Meanwhile, 73 out of the 146 patients had medical records of their preoperative fecal elastase-1 levels to assess exocrine pancreatic function. All of the samples were collected 4 days preoperatively. The fecal elastase-1 concentrations were measured using a commercially available enzyme-linked immunoassay (ELISA) kit (Schebo Biotech AG, Giessen, Germany).

### Definition of POPF

Pancreatic fistula was defined by output via an operative drain of any measurable volume of drain fluid on or after postoperative day 3 with amylase content greater than three times the upper normal serum level (more than 300 IU/L) according to the definition given by the International Study Group on Pancreatic Fistula (ISGPF) [24]. A grade A fistula is transient and asymptomatic, evident only by elevated drain amylase levels. A grade B leakage necessitates changes in patient management or adjustments in the clinical treatment plan, including the introduction of antibiotic therapy, supplemental nutrition, somatostatin analogs, and percutaneous drainage. A grade C fistula is the most severe and necessitates major deviations in the clinical management. Furthermore, a grade C fistula might result in sepsis, organ dysfunction, and even death and may require surgical exploration for definitive management. For the purpose of this study, the patients were divided into two groups: non- POPF group (n = 107, 73%) and POPF group (n = 39, 27%) including grade A (n = 26), grade B (n = 9) and grade C (n = 4).

### CT protocol

For the 86 out of 146 patients, preoperative dynamic CT was performed using one of two scanners: a 16-slice multidetector CT (MDCT) scanner (Somatom Sensation 16, Siemens Medical Solutions, Erlangen, Germany), or a 64-slice MDCT scanner (Somatom Sensation 64, Siemens Medical Solutions, Erlangen, Germany). All the patients were instructed to fast for at least 5 hours before they underwent CT examination. Each patient was administered 150 mL of a nonionic contrast material (Ultravist 300, Schering AG, Berlin, Germany) intravenously by means of a power injector (EnVision CT, Medrad, Pittsburgh, Pa) at a rate of 3 mL/second. The CT images were acquired in a craniocaudal direction with the following parameters: detector collimation, 16 × 0.75 mm; table feed, 12 mm per rotation; section width, 3 mm; reconstruction increment, 3 mm with 3-mm-thick sections; pitch, 1.2; tube voltage, 120 kVp; and tube current, 160 mAs. Precontrast (Pre) scanning (i.e., the first pass) was performed first, followed by contrast-enhanced CT. In order to determine the time of peak aortic enhancement, a bolus injection of 20 mL of contrast material was administered, and sequential dynamic sections were acquired every 2 seconds, starting from the hepatic hilum. Based on the findings of a previous study on multidetector row helical CT, we calculated the start time for the arterial phase (AP) by adding 15 seconds to the time of peak aortic enhancement calculated at the hepatic hilum. The ensuing average start time for the arterial phase was 34 seconds (range, 30–38 seconds). The portal venous phase scan was acquired at 70 seconds after the start of the contrast material injection. The equilibrium phase (EP) scan was acquired at 3 minutes after the start of the contrast material injection. Since our institute was a tertiary referral hospital, and there were many patients referred here from other institutes, CT data of other 60 patients were acquired at other hospitals using non-unified dynamic imaging protocols.

### Data analysis

**Fig 1:**
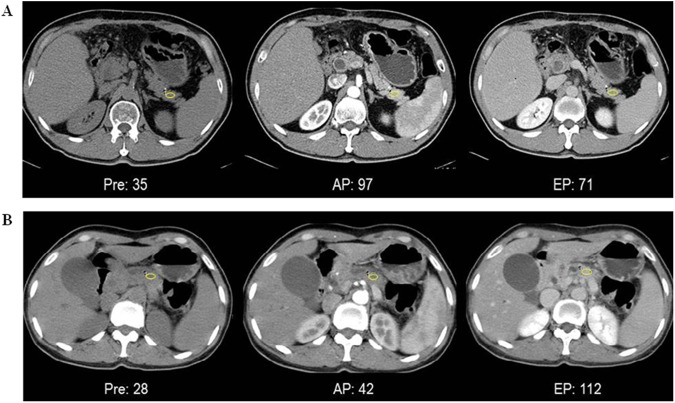
Measurement of pancreatic parenchymal attenuation density with ROI (region of interest). A, B. Comparing to normal pancreatic parenchyma (A), fibrotic pancreas (B) shows gradually increased and higher attenuation densities at the same ROI. Pre, pre-contrast; AP, arterial phase; EP, equilibrium phase.

Two radiologists, blinded to the patients' clinical data, analyzed the preoperative CT images. The CT attenuation values of the pancreatic parenchyma were measured on the unenhanced images and the images obtained in the arterial and equilibrium phases after contrast administration, by placing the ROI in the pancreas over the superior mesenteric vein in an area unaffected by the tumor. The largest possible circular or oval ROI was placed with every effort to avoid the pancreatic mass, pancreatic duct, and partial volume averaging from the extrapancreatic structures. The smallest ROI was approximately 3 mm in diameter in cases where the pancreatic parenchyma was atrophic. The mean value of the CT attenuations were calculated, and the three attenuation parameters were calculated as follows: (AP–Pre)/Pre, (EP–Pre)/Pre and (EP–Pre)/(AP–Pre) (Fig 1).

### Statistical analysis

The mean CT attenuation values and fecal elastase-1 levels of the patients with POPF (grade A, B, or C) and those of the patients without POPF were compared using the independent two-sample *t*-test. Logistic regression models were used to evaluate the risk factors for the development of POPF for univariate analysis. To evaluate the efficacy of the risk factors in the prediction of POPF development and to establish the optimal cut-off points, the receiver operating characteristic (ROC) curve and Youden analyses were performed. The ROC comparison was performed to compare the ability of the clinical cut-off value of fecal elastase-1 to discriminate the POPF from non-POPF cases with that of the cut-off value obtained using the Youden method. For the combined assessment of the ability of the CT and fecal elastase-1 findings to predict POPF development, the ROC comparison and tree analysis were performed. A p-value <0.05 was considered statistically significant. All of the statistical calculations were performed using the SAS software (release 9.2; SAS Institute Inc., Cary, NC, USA).

## Results

**Fig 2:**
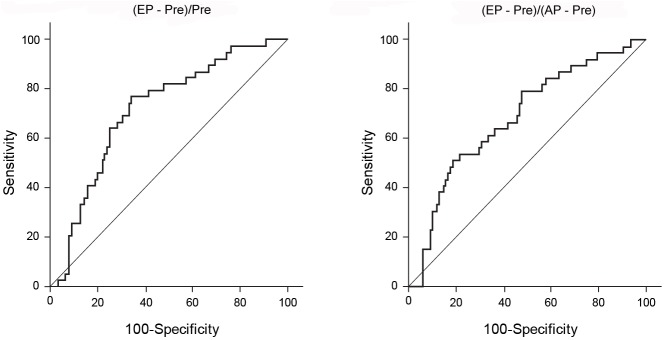
Receiver operating characteristic (ROC) curve of the CT attenuation ratios. Pre, pre-contrast; AP, arterial phase; EP, equilibrium phase.

The mean values of (AP–Pre)/Pre show a decreasing trend with the increase in the grade of POPF (Table 1). The mean values of (EP–Pre)/Pre and (EP–Pre)/(AP–Pre) also show a decreasing trend with the increase in the grade of POPF (Table 1). In the non-POPF group, the pancreas showed delayed enhancement compared to the pancreatic enhancement pattern seen in the POPF group. Therefore, the mean values of (EP–Pre)/Pre and (EP–Pre)/(AP–Pre) of the non-POPF group were significantly higher than those of the POPF group (2.26±3.63 vs. 1.04±0.51; p = 0.001 and 1.12±1.40 vs. 0.71±0.29; p = 0.006, respectively). In logistic regression analyses, the values of (EP–Pre)/Pre and (EP–Pre)/(AP–Pre) were found to be significant predictors for the development of pancreatic fistula (odds ratio (OR) = 0.243, p = 0.002 and OR = 0.176, p = 0.014, respectively). The optimal cut-off point of (EP–Pre)/Pre for the discrimination of the patients developing POPF from those without POPF was determined to be 1.100 (areas under the curve [AUC], 0.749; sensitivity, 77%; and specificity, 69%) and that of (EP–Pre)/(AP–Pre) was determined to be 0.60 (AUC, 0.684; sensitivity, 51%; and specificity, 81%) (Fig 2).

**Table 1:** Mean values of the three CT attenuation ratios according to the POPF grade.

Calculated parameters	Non-POPF	Grade A	Grade B	Grade C	Mean values
	(n = 107)	(n = 26)	(n = 9)	(n = 4)	
(AP–Pre)/Pre	2.08 ± 1.53	1.56 ± 0.80	1.59 ± 0.47	1.24 ± 0.33	1.93 ± 1.38
(EP–Pre)/Pre	2.26 ± 3.63	1.02 ± 0.55	1.19 ± 0.47	0.82 ± 0.20	1.93 ± 3.16
(EP–Pre)/(AP–Pre)	1.12 ± 1.40	0.70 ± 0.30	0.77 ± 0.35	0.67 ± 0.11	1.01 ± 1.22

AP, arterial phase; Pre, pre-contrast; EP, equilibrium phase.

**Fig 3:**
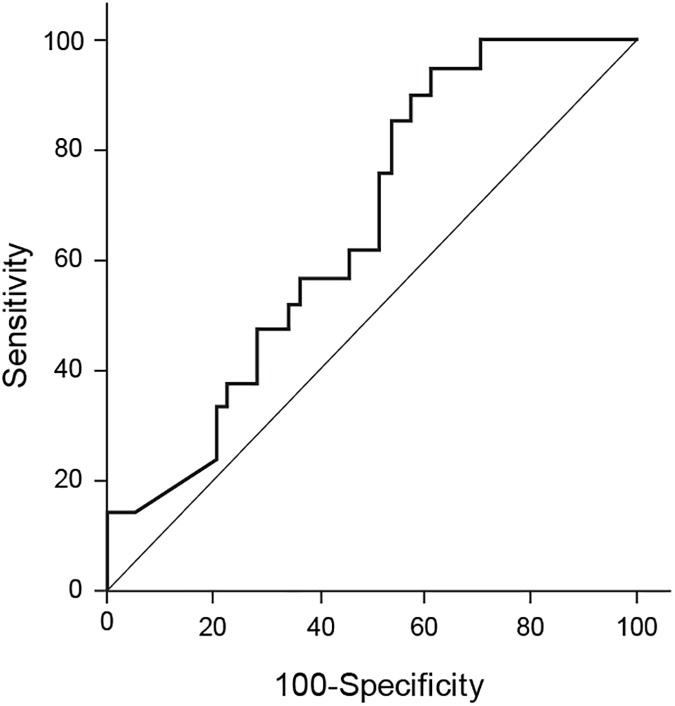
ROC curve of the fecal elastase-1 levels.

**Fig 4:**
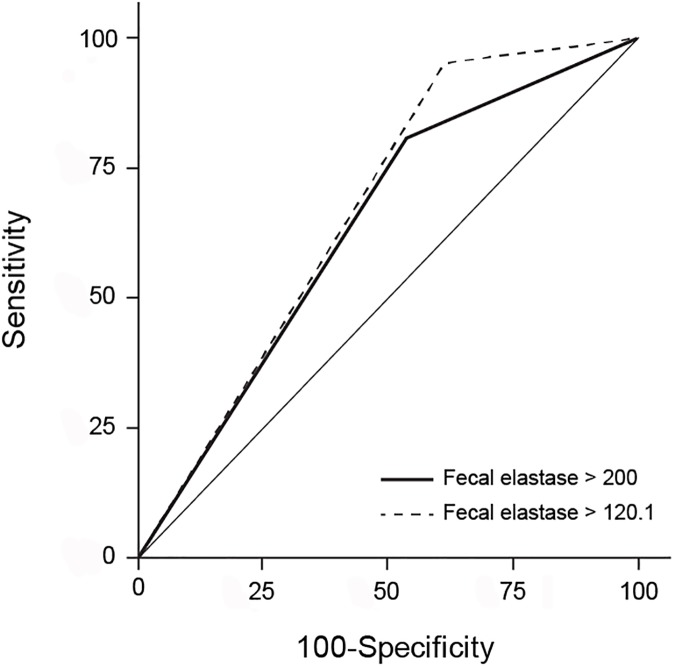
ROC curve for the comparison of the fecal elastase-1 levels at the cut-off points of 120.1μg/g stool and 200 μg/g stool.

Of the 73 patients whose records of preoperative fecal elastase-1 levels were available, 21 (29%) developed POPF (grade A, 15; grade B, 4; and grade C, 2). The non-POPF group was composed of the remaining 52 patients. The mean preoperative fecal elastase-1 level of the POPF group was significantly higher than that of the non-POPF group (359.4 vs. 252.0; p *=* 0.029). In the logistic regression analyses, fecal elastase-1 was a marginally meaningful predictive factor of pancreatic fistula (OR = 1.003, p = 0.034). According to the ROC curve and Youden analyses, the AUC was 0.657 (95% CI, 0.536–0.764) and the optimal cut-off point for the discrimination of the patients with POPF from those without POPF was 120.1 (sensitivity, 95%; specificity, 38%) (Fig 3). Clinically, fecal elastase-1 values greater than 200μg/g stool indicate normal exocrine pancreatic function and those ≤ 200μg/g stool suggest exocrine pancreatic insufficiency [22]. In the ROC comparison of the ability of the clinical cut-off value (200μg/g stool) and that of the cut-off value obtained using the Youden method (120μg/g stool), to discriminate the patients with POPF from those without, the value of AUC was slightly higher when the cut-off point was 120μg/g stool; however, there was no statistically significant difference between the predictive abilities of the two (AUC = 0.669 for cut-off point = 120μg/g stool; AUC = 0.636 for cut-off point = 200μg/g stool; p = 0.447) (Fig 4).

**Fig 5:**
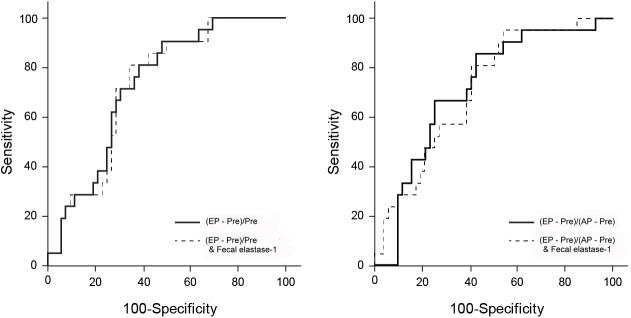
ROC curve for the comparison of the abilities of the CT enhancement ratios alone and the combination of CT enhancement ratios and fecal elastase-1 levels to predict postoperative pancreatic fistulae. Pre, pre-contrast; AP, arterial phase; EP, equilibrium phase.

Of the 73 patients with available preoperative dynamic CT and fecal elastase-1 level records, the AUC values of the stand-alone CT enhancement ratios were slightly higher than those of the combination of the CT enhancement ratios and fecal elastase-1 levels; however, there were no statistically significant difference (AUC = 0.729 for (EP–Pre)/Pre; AUC = 0.726 for the combination of (EP–Pre)/Pre and fecal elastase-1 levels; p = 0.897 and AUC = 0.720 for (EP–Pre)/(AP–Pre); AUC = 0.716 for the combination of (EP–Pre)/(AP–Pre) and fecal elastase-1 levels; p = 0.917) (Fig 5).

**Fig 6:**
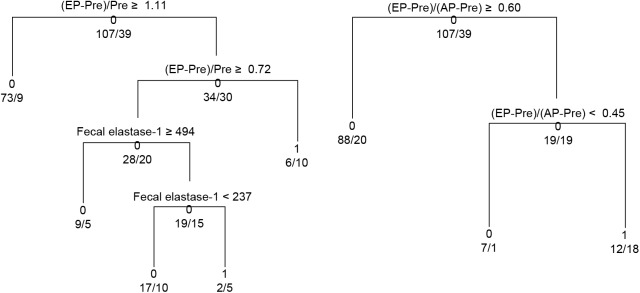
Tree analysis of the abilities of the CT enhancement ratios and fecal elastase-1 levels to predict postoperative pancreatic fistulae. Pre, pre-contrast; AP, arterial phase; EP, equilibrium phase.

A tree analysis was performed to evaluate the efficacy of the CT enhancement ratios and fecal elastase-1 levels for the prediction of POPF and to establish their cut- off values when evaluated in conjunction (Fig 6). In the tree analysis of the (EP–Pre)/Pre values and fecal elastase-1 levels on conjunction, the CT enhancement ratio was the more powerful tool for the prediction of POPF. At (EP–Pre)/Pre values ≥1.11, the non-occurrence of POPF could be expected regardless of the fecal elastase-1 levels; at (EP–Pre)/Pre values less than 0.72, POPF could be expected to develop regardless of the fecal elastase-1 levels; and at (EP–Pre)/Pre values between 0.72 and 1.11, fecal elastase-1 levels could help predict the development of POPF. In the tree analysis of the (EP–Pre)/(AP–Pre) values and fecal elastase-1 levels in conjuction, the efficacy of the latter for POPF prediction could be ignored. At (EP–Pre)/(AP–Pre) values >0.60, the non-occurrence of POPF could be expected regardless of the fecal elastase-1 levels.

## Discussion

The mortality rate associated with pancreatoduodenectomy has decreased to less than 5% due to advances in the postoperative care and surgical techniques [5,12,25–32]. However, the morbidity still remains high, with a rate of 30–60%, and is often related to the occurrence of POPF [6–8]. The risk factors for the development of pancreatic fistula after PD have been extensively studied [5–10,12,25,26,28,30–33]. The presence of soft pancreatic parenchyma is the most widely recognized of those risk factors [5,9,12]. On the other hand, the presence of pancreatic fibrosis with a firm pancreatic texture is thought to decrease the risk of POPF development [4–18,34]. Therefore, the preoperative assessment of pancreatic fibrosis might be helpful in predicting POPF and in pre- and post-operative management. When identifying high risk patients for pancreatic fistula accurately and easily with preoperative assessment of pancreatic fibrosis, protocols for administration of perioperative pasireotide, a somatostatin analogue that decreases the rate of clinically significant pancreatic fistula, would be clearly guided to maximizing cost-efficacy in high risk patients [35, 36].

Recent studies have shown that CT or MRI perfusion imaging can detect the microcirculatory changes caused by collagen deposition in the pancreas [14–16,18]. These studies have shown that pancreatic fibrosis is characterized by delayed enhancement with a slow rise to the peak, followed by a slow decline or plateau, while the normal pancreas shows a rapid rise to the peak, followed by a rapid decline. Only a few studies have described the quantitative assessment of the pancreatic enhancement characteristics on the preoperative CT or MRI, as a method to estimate the risk of developing POPF. Dinter et al. calculated a muscle-normalized signal intensity (SI) curve with a SI ratio using dynamic contrast-enhanced MRI in 72 patients who underwent PD by duct-to-mucosa pancreatojejunostomy [14]. The SI ratios were grouped into two groups: rapid increase (SI ratio ≥1.1; early arterial value > portal-venous value; soft pancreas) and equilibrium increase (SI ratio <1.1; firm or hard pancreas) groups. In multivariate analysis, the SI ratio ≥1.1 was the only preoperative parameter that could predict the leakage. Using a dual-phase pancreatic CT technique, Hashimoto et al. expressed the late phase/early phase ratio (L/E ratio), calculated as (hepatic phase—unenhanced phase)/(pancreatic phase—unenhanced phase), to indicate delayed phase enhancement [37]. The L/E ratio and histological grade of pancreatic fibrosis were correlated with the development of a clinically relevant pancreatic anastomotic failure (PAF) and the other clinical parameters. In the multivariate analyses, the L/E ratio and body mass index (BMI) were found to be significant predictors for the development of a clinically relevant PAF.

In the present study, we expected that the mean value of (AP–Pre)/Pre of the POPF group would be higher than that of the non-POPF group. However, the actual result obtained was different from what was expected. Commonly, the pancreatic phase scans are acquired 40–70 seconds after the infusion of the intravenous contrast material, at which time, the tumor-to-pancreas attenuation difference is the greatest [38,39]. In the previous studies, that evaluated the enhancement patterns of the normal pancreas and the fibrotic pancreas with autoimmune pancreatitis or chronic pancreatitis, the early images of the pancreatic enhancement peak were obtained 25–45 seconds after the administration of the contrast material [15,17,18,40,41]. In this narrow time range of barely dozens of seconds, the time-attenuation curve showed a steep slope. Therefore, it is possible that the values of pancreatic attenuation vary significantly with the time differences of just a few seconds during the narrowing of the time range. Furthermore, in the study of Tajima et al., the time-signal intensity curve (TIC) of the fibrotic pancreas showed a slow rise to the peak value at 1–2 min after the administration of contrast material [17]. The recruited patients in the present study included a number of patients referred from other hospitals, which caused discrepancies in the obtaining of the early phase images for the dynamic imaging protocol. It was, therefore, difficult to measure the attenuation value of the pancreas at the arterial phase in all of the cases at a standardized time. Given this situation, it was difficult to obtain as reliable result as was expected using the arterial image data. Meanwhile, in order to express the delayed enhancement corresponding to pancreatic fibrosis, we used a 3-min delayed CT attenuation value after contrast administration, and calculated two enhancement ratios, (EP–Pre)/Pre and (EP–Pre)/(AP–Pre), while previous studies mentioned above used CT attenuation values of hepatic or portal-venous phases 60–70 sec after the administration of the contrast material [14,37]. In most studies, the time-attenuation curve showed a plateau or gradual slope during the 3-min delay after contrast administration with higher attenuation values in the fibrotic pancreas compared to those in the normal pancreas [15,17,18,40,41]. Therefore, there would be no remarkable differences between the pancreatic attenuation values based on the difference of a few or dozens of seconds. Furthermore, in the various dynamic CT protocols of different hospitals, there were slight differences in the images of the AP or portal venous phases, but the EP images were acquired approximately 3 min after contrast administration, in most cases. Therefore, using the 3-min delayed CT attenuation values to express the delayed enhancement corresponding to pancreatic fibrosis seems reasonable in practice. Pancreatic fibrosis causes the loss of the exocrine function of the pancreas. There have been several studies reporting that a soft pancreas has a good exocrine function and secretes more pancreatic juices, which contain proteolytic enzymes [42,43]. Therefore, the exocrine pancreatic function would most probably be correlated with pancreatic fibrosis, and could be used to preoperatively predict POPF development. Human fecal elastase-1 is a pancreas-specific proteolytic enzyme, not degraded during intestinal transport and reaches concentrations in feces 5–6 times those in the duodenal juice, and has been proposed as a suitable marker for pancreatic insufficiency [21–23]. The measurement of fecal elastase-1 levels by ELISA is sensitive, specific, and noninvasive [44,45]. In the present study, the fecal elastase-1 level was a statistically significant predictive factor of pancreatic fistula; but, there were no great differences in the AUC values between the CT enhancement ratios and fecal elastase-1 levels in conjunction and the CT enhancement ratios alone. This indicates that there is no added benefit in using the CT enhancement ratios in conjunction with the fecal elastase-1 levels for the discrimination of the patients with POPF from those without. It is thought that these results are probably related to the relatively low odd ratios of the fecal elastase-1 levels.

There were some limitations to this study. Firstly, because of the retrospective nature of our study, there were no reference standards of the pathological fibrosis data to correlate with the CT enhancement ratios or fecal elastase-1 levels for the validation of our results. Secondly, the number of patients with the records of fecal elastase-1 levels available was quite small; this might be a reason for the low odd ratios of fecal elastase-1 levels, which resulted in a negligible combined predictive value of the two preoperative predictive tools of POPF. Thirdly, because postoperative pancreatic stent (P-J stent) was routinely used in all subjected patients, we did not concern the effect of the stent related to the incidence of POPF. We just presumed that low incidence of POPF in the recruited patients’ group in the present study might be related to the use of postoperative P-J stent [46]. With the low incidence of post-operative pancreatic fistula, the value of fecal elastase-1 might be rather limited comparing to the prior reports; however, there were no controlled data for the use of P-J stents, the relationship between the P-J stent and pre-operative fecal elastase could not be discussed here. Finally, the number of patients with grade C POPF was too small to separate the analysis of the correlation between the CT enhancement ratios/fecal elastase-1 levels and the POPF grades according to the clinical severity. However, we showed the decreasing trend of the mean values of the CT enhancement ratios according to the POPF grade.

In conclusion, depending on the relationship between the delayed enhancement of the pancreatic parenchyma on dynamic CT and the absence of POPF without remarkable added benefit of combining the fecal elastase-1 levels, CT enhancement ratios acquired at EP regardless of fecal elastase-1 data can be used as a reliable predictor of the risk of developing a POPF.

## Supporting information

S1 File Patients’ data of mean hounsfield numbers of pancreatic parenchyma on pre-operative dynamic CT and pre-operative fecal elastase-1. Fistula grade was defined by the International Study Group on Pancreatic Fistula (ISGPF) [24]. Pre, pre-contrast; AP, arterial phase; EP, equilibrium phase; Elastase, fecal elastase-1.
(XLS)

## References

[1] Richter A, Niedergethmann M, Sturm JW, Lorenz D, Post S, Trede M. Long-term results of partial pancreaticoduodenectomy for ductal adenocarcinoma of the pancreatic head: 25-year experience. World J Surg 2003;27:324–9. doi: 10.1007/s00268-002-6659-z 12607060 10.1007/s00268-002-6659-z

[2] Trede M, Schwall G, Saeger HD. Survival after pancreatoduodenectomy. 118 consecutive resections without an operative mortality. Ann Surg 1990;211:447–58. 2322039 10.1097/00000658-199004000-00011 PMC1358031

[3] Adam U, Makowiec F, Riediger H, Schareck WD, Benz S, Hopt UT. Risk factors for complications after pancreatic head resection. Am J Surg 2004;187:201–8. doi: 10.1016/j.amjsurg.2003.11.004 14769305 10.1016/j.amjsurg.2003.11.004

[4] DeOliveira ML, Winter JM, Schafer M, Cunningham SC, Cameron JL, Yeo CJ, et al Assessment of complications after pancreatic surgery: A novel grading system applied to 633 patients undergoing pancreaticoduodenectomy. Ann Surg 2006;244:931–7; discussion 7–9. doi: 10.1097/01.sla.0000246856.03918.9a 17122618 10.1097/01.sla.0000246856.03918.9a PMC1856636

[5] Hashimoto Y, Traverso LW. Incidence of pancreatic anastomotic failure and delayed gastric emptying after pancreatoduodenectomy in 507 consecutive patients: Use of a web-based calculator to improve homogeneity of definition. Surgery 2010;147:503–15. doi: 10.1016/j.surg.2009.10.034 20018335 10.1016/j.surg.2009.10.034

[6] Murakami Y, Uemura K, Hayashidani Y, Sudo T, Hashimoto Y, Nakagawa N, et al No mortality after 150 consecutive pancreatoduodenctomies with duct-to-mucosa pancreaticogastrostomy. J Surg Oncol 2008;97:205–9. doi: 10.1002/jso.20903 18050288 10.1002/jso.20903

[7] Poon RT, Fan ST, Lo CM, Ng KK, Yuen WK, Yeung C, et al External drainage of pancreatic duct with a stent to reduce leakage rate of pancreaticojejunostomy after pancreaticoduodenectomy: a prospective randomized trial. Ann Surg 2007;246:425–33; discussion 33–5. doi: 10.1097/SLA.0b013e3181492c28 17717446 10.1097/SLA.0b013e3181492c28 PMC1959348

[8] Pratt WB, Maithel SK, Vanounou T, Huang ZS, Callery MP, Vollmer CM Jr. Clinical and economic validation of the International Study Group of Pancreatic Fistula (ISGPF) classification scheme. Ann Surg 2007;245:443–51. doi: 10.1097/01.sla.0000251708.70219.d2 17435552 10.1097/01.sla.0000251708.70219.d2 PMC1877022

[9] Lin JW, Cameron JL, Yeo CJ, Riall TS, Lillemoe KD. Risk factors and outcomes in postpancreaticoduodenectomy pancreaticocutaneous fistula. J Gastrointest Surg 2004;8:951–9. doi: 10.1016/j.gassur.2004.09.044 15585382 10.1016/j.gassur.2004.09.044

[10] Hashimoto Y, Traverso LW. Pancreatic anastomotic failure rate after pancreaticoduodenectomy decreases with microsurgery. J Am Coll Surg 2010;211:510–21. doi: 10.1016/j.jamcollsurg.2010.06.018 20801693 10.1016/j.jamcollsurg.2010.06.018

[11] Yeo CJ, Cameron JL, Lillemoe KD, Sauter PK, Coleman J, Sohn TA, et al Does prophylactic octreotide decrease the rates of pancreatic fistula and other complications after pancreaticoduodenectomy? Results of a prospective randomized placebo-controlled trial. Ann Surg 2000;232:419–29. 10973392 10.1097/00000658-200009000-00014 PMC1421155

[12] Yeo CJ, Cameron JL, Maher MM, Sauter PK, Zahurak ML, Talamini MA, et al A prospective randomized trial of pancreaticogastrostomy versus pancreaticojejunostomy after pancreaticoduodenectomy. Ann Surg 1995;222:580–8; discussion 8–92. 7574936 10.1097/00000658-199510000-00014 PMC1234894

[13] Nakamura H, Murakami Y, Uemura K, Hayashidani Y, Sudo T, Ohge H, et al Predictive factors for exocrine pancreatic insufficiency after pancreatoduodenectomy with pancreaticogastrostomy. J Gastrointest Surg 2009;13:1321–7. doi: 10.1007/s11605-009-0896-5 19415402 10.1007/s11605-009-0896-5

[14] Dinter DJ, Aramin N, Weiss C, Singer C, Weisser G, Schoenberg SO, et al Prediction of anastomotic leakage after pancreatic head resections by dynamic magnetic resonance imaging (dMRI). J Gastrointest Surg 2009;13:735–44. doi: 10.1007/s11605-008-0765-7 19057965 10.1007/s11605-008-0765-7

[15] Kim T, Murakami T, Takamura M, Hori M, Takahashi S, Nakamori S, et al Pancreatic mass due to chronic pancreatitis: correlation of CT and MR imaging features with pathologic findings. AJR Am J Roentgenol 2001;177:367–71. doi: 10.2214/ajr.177.2.1770367 11461864 10.2214/ajr.177.2.1770367

[16] Lee SE, Jang JY, Lim CS, Kang MJ, Kim SH, Kim MA, et al Measurement of pancreatic fat by magnetic resonance imaging: predicting the occurrence of pancreatic fistula after pancreatoduodenectomy. Ann Surg 2010;251:932–6. doi: 10.1097/SLA.0b013e3181d65483 20395858 10.1097/SLA.0b013e3181d65483

[17] Tajima Y, Matsuzaki S, Furui J, Isomoto I, Hayashi K, Kanematsu T. Use of the time-signal intensity curve from dynamic magnetic resonance imaging to evaluate remnant pancreatic fibrosis after pancreaticojejunostomy in patients undergoing pancreaticoduodenectomy. Br J Surg 2004;91:595–600. doi: 10.1002/bjs.4461 15122611 10.1002/bjs.4461

[18] Takahashi N, Fletcher JG, Hough DM, Fidler JL, Kawashima A, Mandrekar JN, et al Autoimmune pancreatitis: differentiation from pancreatic carcinoma and normal pancreas on the basis of enhancement characteristics at dual-phase CT. AJR Am J Roentgenol 2009;193:479–84. doi: 10.2214/AJR.08.1883 19620446 10.2214/AJR.08.1883

[19] Niedergethmann M, Farag Soliman M, Post S. Postoperative complications of pancreatic cancer surgery. Minerva Chir 2004;59:175–83. 15238891

[20] Murakami H, Suzuki H, Nakamura T. Pancreatic fibrosis correlates with delayed gastric emptying after pylorus-preserving pancreaticoduodenectomy with pancreaticogastrostomy. Ann Surg 2002;235:240–5. 11807364 10.1097/00000658-200202000-00012 PMC1422420

[21] Glasbrenner B, Schon A, Klatt S, Beckh K, Adler G. Clinical evaluation of the faecal elastase test in the diagnosis and staging of chronic pancreatitis. Eur J Gastroenterol Hepatol 1996;8:1117–20. 8944376 10.1097/00042737-199611000-00016

[22] Loser C, Mollgaard A, Folsch UR. Faecal elastase 1: a novel, highly sensitive, and specific tubeless pancreatic function test. Gut 1996;39:580–6. 8944569 10.1136/gut.39.4.580 PMC1383273

[23] Stein J, Jung M, Sziegoleit A, Zeuzem S, Caspary WF, Lembcke B. Immunoreactive elastase I: clinical evaluation of a new noninvasive test of pancreatic function. Clin Chem 1996;42:222–6. 8595714

[24] Bassi C, Dervenis C, Butturini G, Fingerhut A, Yeo C, Izbicki J, et al Postoperative pancreatic fistula: an international study group (ISGPF) definition. Surgery 2005;138:8–13. doi: 10.1016/j.surg.2005.05.001 16003309 10.1016/j.surg.2005.05.001

[25] Cameron JL, Pitt HA, Yeo CJ, Lillemoe KD, Kaufman HS, Coleman J. One hundred and forty-five consecutive pancreaticoduodenectomies without mortality. Ann Surg 1993;217:430–5; discussion 5–8. 8098202 10.1097/00000658-199305010-00002 PMC1242815

[26] Farnell MB, Pearson RK, Sarr MG, DiMagno EP, Burgart LJ, Dahl TR, et al A prospective randomized trial comparing standard pancreatoduodenectomy with pancreatoduodenectomy with extended lymphadenectomy in resectable pancreatic head adenocarcinoma. Surgery 2005;138:618–28; discussion 28–30. doi: 10.1016/j.surg.2005.06.044 16269290 10.1016/j.surg.2005.06.044

[27] Lillemoe KD, Cameron JL, Kim MP, Campbell KA, Sauter PK, Coleman JA, et al Does fibrin glue sealant decrease the rate of pancreatic fistula after pancreaticoduodenectomy? Results of a prospective randomized trial. J Gastrointest Surg 2004;8:766–72; discussion 72–4. doi: 10.1016/j.gassur.2004.06.011 15531229 10.1016/j.gassur.2004.06.011

[28] Lowy AM, Lee JE, Pisters PW, Davidson BS, Fenoglio CJ, Stanford P, et al Prospective, randomized trial of octreotide to prevent pancreatic fistula after pancreaticoduodenectomy for malignant disease. Ann Surg 1997;226:632–41. 9389397 10.1097/00000658-199711000-00008 PMC1191125

[29] Marcus SG, Cohen H, Ranson JH. Optimal management of the pancreatic remnant after pancreaticoduodenectomy. Ann Surg 1995;221:635–45; discussion 45–8. 7794068 10.1097/00000658-199506000-00003 PMC1234686

[30] Pedrazzoli S, Liessi G, Pasquali C, Ragazzi R, Berselli M, Sperti C. Postoperative pancreatic fistulas: preventing severe complications and reducing reoperation and mortality rate. Ann Surg 2009;249:97–104. doi: 10.1097/SLA.0b013e31819274fe 19106683 10.1097/SLA.0b013e31819274fe

[31] Roder JD, Stein HJ, Bottcher KA, Busch R, Heidecke CD, Siewert JR. Stented versus nonstented pancreaticojejunostomy after pancreatoduodenectomy: a prospective study. Ann Surg 1999;229:41–8. 9923798 10.1097/00000658-199901000-00005 PMC1191606

[32] Sarr MG. The potent somatostatin analogue vapreotide does not decrease pancreas-specific complications after elective pancreatectomy: a prospective, multicenter, double-blinded, randomized, placebo-controlled trial. J Am Coll Surg 2003;196:556–64; discussion 64–5; author reply 65. doi: 10.1016/S1072-7515(03)00104-2 12691930 10.1016/S1072-7515(03)00104-2

[33] Friess H, Malfertheiner P, Isenmann R, Kuhne H, Beger HG, Buchler MW. The risk of pancreaticointestinal anastomosis can be predicted preoperatively. Pancreas 1996;13:202–8. 8829190

[34] Hamanaka Y, Nishihara K, Hamasaki T, Kawabata A, Yamamoto S, Tsurumi M, et al Pancreatic juice output after pancreatoduodenectomy in relation to pancreatic consistency, duct size, and leakage. Surgery 1996;119:281–7. 8619183 10.1016/s0039-6060(96)80114-0

[35] Allen PJ. Pasireotide for postoperative pancreatic fistula. N Engl J Med 2014;370:2014–2022. doi: 10.1056/NEJMoa1313688 24849084 10.1056/NEJMoa1313688

[36] Ma LW. The Cost of Postoperative Pancreatic Fistula Versus the Cost of Pasireotide: Results from a Prospective Randomized Trial. Ann Surg 2016. 10.1097/SLA.0000000000001892 PMC5182113 27429029

[37] Hashimoto Y, Sclabas GM, Takahashi N, Kirihara Y, Smyrk TC, Huebner M, et al Dual-phase computed tomography for assessment of pancreatic fibrosis and anastomotic failure risk following pancreatoduodenectomy. J Gastrointest Surg 2011;15:2193–204. doi: 10.1007/s11605-011-1687-3 21948179 10.1007/s11605-011-1687-3

[38] Lu DS, Vedantham S, Krasny RM, Kadell B, Berger WL, Reber HA. Two-phase helical CT for pancreatic tumors: pancreatic versus hepatic phase enhancement of tumor, pancreas, and vascular structures. Radiology 1996;199:697–701. doi: 10.1148/radiology.199.3.8637990 8637990 10.1148/radiology.199.3.8637990

[39] Fletcher JG, Wiersema MJ, Farrell MA, Fidler JL, Burgart LJ, Koyama T, et al Pancreatic malignancy: value of arterial, pancreatic, and hepatic phase imaging with multi-detector row CT. Radiology 2003;229:81–90. doi: 10.1148/radiol.2291020582 14519871 10.1148/radiol.2291020582

[40] Irie H, Honda H, Baba S, Kuroiwa T, Yoshimitsu K, Tajima T, et al Autoimmune pancreatitis: CT and MR characteristics. American Journal of Roentgenology 1998;170:1323–7. doi: 10.2214/ajr.170.5.9574610 9574610 10.2214/ajr.170.5.9574610

[41] Kawamoto S, Siegelman SS, Hruban RH, Fishman EK. Lymphoplasmacytic Sclerosing Pancreatitis with Obstructive Jaundice: CT and Pathology Features. American Journal of Roentgenology 2004;183:915–21. doi: 10.2214/ajr.183.4.1830915 15385281 10.2214/ajr.183.4.1830915

[42] Suzuki Y, Fujino Y, Tanioka Y, Hiraoka K, Takada M, Ajiki T, et al Selection of pancreaticojejunostomy techniques according to pancreatic texture and duct size. Arch Surg 2002;137:1044–7; discussion 8. 12215157 10.1001/archsurg.137.9.1044

[43] Sato N, Yamaguchi K, Chijiiwa K, Tanaka M. Risk analysis of pancreatic fistula after pancreatic head resection. Arch Surg 1998;133:1094–8. 9790207 10.1001/archsurg.133.10.1094

[44] Sziegoleit A, Linder D. Studies on the sterol-binding capacity of human pancreatic elastase 1. Gastroenterology 1991;100:768–74. 1993499 10.1016/0016-5085(91)80024-4

[45] Katschinski M, Schirra J, Bross A, Goke B, Arnold R. Duodenal secretion and fecal excretion of pancreatic elastase-1 in healthy humans and patients with chronic pancreatitis. Pancreas 1997;15:191–200. 9260205 10.1097/00006676-199708000-00012

[46] Dong Z, Xu J, Wang Z, Petrov MS. Stents for the prevention of pancreatic fistula following pancreaticoduodenectomy. Cochrane Database Syst Rev 2016 5 6;(5):CD008914 Review. doi: 10.1002/14651858.CD008914.pub3 27153248 10.1002/14651858.CD008914.pub3 PMC7156907

